# Using Coarse-Grained Simulations to Characterize the Mechanisms of Protein–Protein Association

**DOI:** 10.3390/biom10071056

**Published:** 2020-07-15

**Authors:** Kalyani Dhusia, Zhaoqian Su, Yinghao Wu

**Affiliations:** Department of Systems and Computational Biology, Albert Einstein College of Medicine,1300 Morris Park Avenue, Bronx, NY 10461, USA; kalyani.dhusia@einsteinmed.org (K.D.); zhaoqian.su@einsteinmed.org (Z.S.)

**Keywords:** coarse-grained simulations, protein-protein association, physics-based force field, statistical potential

## Abstract

The formation of functionally versatile protein complexes underlies almost every biological process. The estimation of how fast these complexes can be formed has broad implications for unravelling the mechanism of biomolecular recognition. This kinetic property is traditionally quantified by association rates, which can be measured through various experimental techniques. To complement these time-consuming and labor-intensive approaches, we developed a coarse-grained simulation approach to study the physical processes of protein–protein association. We systematically calibrated our simulation method against a large-scale benchmark set. By combining a physics-based force field with a statistically-derived potential in the simulation, we found that the association rates of more than 80% of protein complexes can be correctly predicted within one order of magnitude relative to their experimental measurements. We further showed that a mixture of force fields derived from complementary sources was able to describe the process of protein–protein association with mechanistic details. For instance, we show that association of a protein complex contains multiple steps in which proteins continuously search their local binding orientations and form non-native-like intermediates through repeated dissociation and re-association. Moreover, with an ensemble of loosely bound encounter complexes observed around their native conformation, we suggest that the transition states of protein–protein association could be highly diverse on the structural level. Our study also supports the idea in which the association of a protein complex is driven by a “funnel-like” energy landscape. In summary, these results shed light on our understanding of how protein–protein recognition is kinetically modulated, and our coarse-grained simulation approach can serve as a useful addition to the existing experimental approaches that measure protein–protein association rates.

## 1. Introduction

Nearly all biological processes in living cells are achieved by the interactions among functionally different proteins [1,2,3,4,5,6]—for instance, the assembly of transient signaling complexes [7,8] or more stable biomolecular machines [9,10,11]. The dynamic properties of protein interactions are not only characterized by the dissociation constants (*K_d_*), which determines the thermodynamic stability of a protein complex [12], but also the association rate (*k_on_*), which measures its kinetics, i.e., how fast this complex can be formed [13,14]. In a real cellular environment, interactions between proteins are often under kinetic, rather than thermodynamic, control [15,16]. For instance, proteins in cell signaling networks often have more than one binding partner that compete with each other. The difference in the speed of binding between these interactions, no matter how strong they are, regulates the dynamics of signal flows in the network. One example is the natural-killer (NK) cell receptor NKG2D (natural-killer group 2, member D) [17]. The receptor recognizes both cellular and viral ligands with the same binding interface, indicating that these ligands must compete with each other for receptor binding when they coexist in the system. As a result, the difference in association rates of receptor binding between cellular and viral ligands directly regulates the NK cytolytic activity [18]. Additionally, mutations at the binding interfaces of protein complexes often alter the rates of their associations and thereby result in severe pathological outcomes [19]. Using the same example of NKG2D, individuals who carry specific mutants that lower the binding specificity of the receptor to the viral ligands are more likely to get infected by the virus [20]. Therefore, studies of the protein–protein association mechanism on a quantitative level are significant for us to understand the dynamics of many cellular activities; furthermore, they have broad impacts on many other fields, such as drug discovery and protein design.

Relative to the current experimental techniques for measuring the rates of protein–protein interactions, such as surface plasma resonance (SPR) [21] and spectroscopic inhibition assay (IASP) [22], computational modeling approaches are not only less time-consuming and labor-intensive, but can also provide mechanistic details that are inaccessible in the laboratory. As a result, a large variety of computational methods have already been developed to study protein–protein association. Some methods can directly predict association rate constants by feeding the structural and chemical features collected from the binding interfaces of protein complexes into machine-learning-based models [23,24]. These models, however, are not able to provide any insights along the pathway of protein–protein association. Methods using physics-based principles, on the contrary, are used to simulate the detailed process of association. Among these methods, all-atom molecular dynamic (MD) simulations based on the explicit solvent model can reveal the complete protein–protein association kinetics [25,26,27,28,29,30,31,32]. Through MD simulation, it was found that the native protein complexes can be associated through a very structurally diverse transition state ensemble in which no more than 20% of native contacts remained [33]. These all-atom simulations, however, are extremely demanding for computational resources and have so far only been successfully applied to a limited number of cases [33,34]. In comparison, the Brownian dynamic (BD) simulations, based on the implicit solvent model, are more computationally efficient and thus are widely used to study protein–protein association [35,36,37,38,39,40,41,42,43,44,45,46,47,48,49,50,51,52,53,54,55,56,57]. In particular, a recent method based on BD simulation and a “transient-complex” theory was proven to be able to successfully predict protein association rates and provide mechanistic insights into the association processes [58,59,60,61].

We have previously also developed a coarse-grained simulation approach to study protein–protein association [62]. Positive correlations have been observed between the experimental measurements and our calculated values of association rates. However, the method has not been used to explore the detailed mechanism of association. In this work, we discussed this possibility by systematically calibrating the simulations against a comprehensive benchmark set. For each complex in the benchmark, a large number of simulation trajectories was carried out. Based on the statistical distributions of these trajectories, we showed that an ensemble of loosely bound encounter complexes were formed around their native conformation, suggesting that the transition states of protein–protein association could be highly diverse on the structural level. The analysis of each individual trajectory further suggested that association could be a dynamic process for searching local binding configurations by repeated dissociation and re-association. We found a correlation between the binding energy used in the simulations and the structural similarity of encounter complexes to their native conformation, implying a “funnel-like” landscape of protein–protein interactions along the pathways of their association. The correlation between experimental and our computationally simulated rates of association became stronger after we introduced a statistical potential into the simulations. Finally, we explored the combination of different criteria for encounter complex formation. In summary, our results provided insights into the common features underlying the general process of protein–protein association. This approach therefore offers quantitative characterizations of the dynamics of protein complex formation and can improve our understanding of the mechanisms of these important biological processes.

## 2. Model and Methods

### 2.1. A Benchmark Set of Protein Complexes for Testing the Simulations of Protein Association

Our benchmark set contains a total number of 62 protein complexes. The experimental data of these complexes, including their atomic structures with the corresponding protein data bank (PDB) IDs, experimentally observed associations and ionic strength used in the measurements, are all publicly accessible. Detailed information about this benchmark set is summarized in Table S1 in the Supplementary Materials. Specifically, these complexes, representing a large variety of protein–protein interactions, such as enzyme/inhibitor, ligand/receptor, regulator/effector and antibody/antigen, are collected from two sources. One is a group of 49 protein complex structures collected by Qin et al., which was used as a training set in our previous study [63]. The second source is from the SKEMPI, which is a comprehensive database that contains not only the absolute values but also the changes in binding constants for wild-type and mutated protein complexes. The most updated version, SKEMPI 2.0, includes data of 345 wild-type protein complexes and their 7085 associated mutants [64]. Among all 345 wild-type protein complexes, 114 contain information on association rates. All these data are available online at https://life.bsc.es/pid/skempi2/. Combining these two sources, excluding the overlapping cases, we obtained a total of 108 entries.

For these entries, we only consider the protein complexes with no more than 500 amino acids in total, and the experimentally measured rate constants within the range from 1.0 × 10^5^ to 1.0 × 10^9^ M^−1^s^−1^, in order to systematically test our simulations in a computationally feasible manner. We further removed the protein complexes with non-consistent experimental data of association rates that were collected from different studies. Most of these studies using SPR or IASP may suffer from a number of technical limitations. Moreover, the protein complexes with atypical association pathways or irregular binding interfaces were also eliminated. For example, the complex formed between transcriptional coactivator CBP/p300 and nuclear receptor p160 (PDB 1KBH) exists as a cooperatively folded helical heterodimer. The association of this type of complex cannot be simulated by our method. They are thus excluded from the study. Consequently, the number of protein complexes in the final benchmark was narrowed down from 108 to 62. Coarse-grained Monte Carlo simulations were carried out on all remaining protein complexes, as described in the next section.

### 2.2. A Coarse-Grained Kinetic Monte Carlo Algorithm for Simulating Protein Association

**Figure 1:**
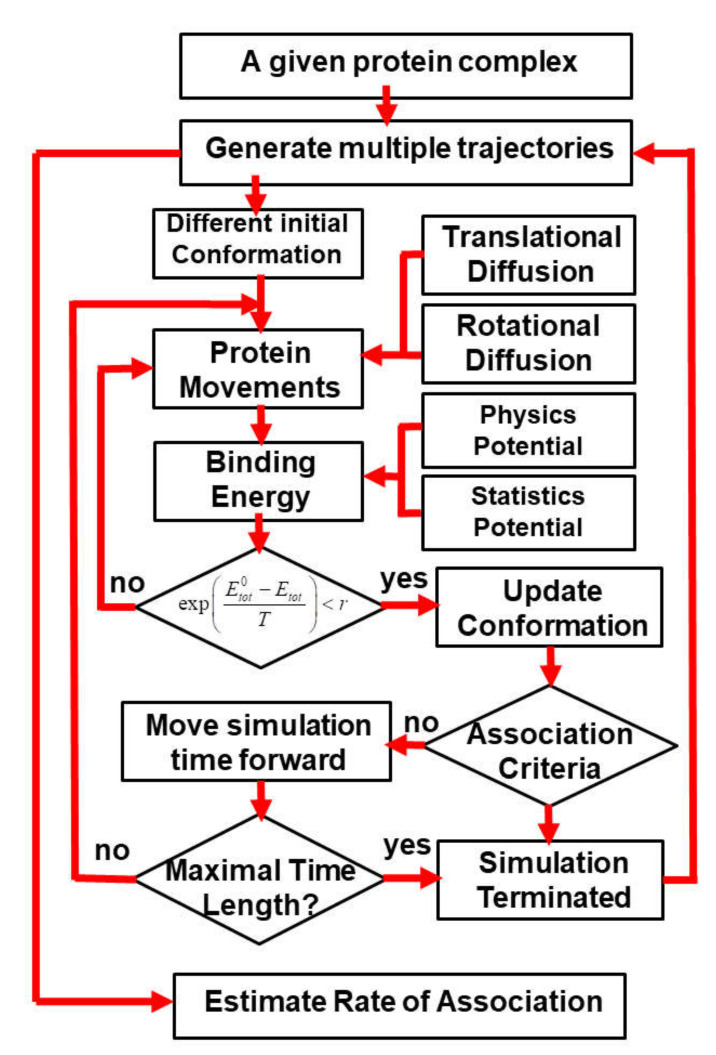
In the kinetic Monte Carlo algorithm, both binding partners in a protein complex undergo random translational and rotational diffusions after an initial random conformation. Diffusions are guided by the intermolecular interactions, which are described by a combination of physics-based force fields and statistically derived potentials. The probability of accepting a new configuration after diffusions is calculated by comparing its energy with the old configuration. If the new configuration is accepted, we further check whether an encounter complex can be formed based on a predefined association criterion. If an encounter complex is formed, the current simulation trajectory will be terminated; otherwise, the simulation continues until it reaches the maximal time duration. Rate of association can be finally estimated by generating a large number of simulation trajectories with this algorithm.

The association of binding partners in a given protein complex is simulated by a previously developed kinetic Monte Carlo (KMC) algorithm [62,65]. The atomic structure of proteins in this method is reduced by a simplified model, in which each residue is coarse-grained into two sites: one is the Cα atom and the other is the functional center of its side-chain. As described in our previous study, the side-chain functional center of a residue is selected based on its specific chemical properties. The simulation is initiated from a random conformation (Figure 1), in which two binding partners of the complex are randomly placed in the three-dimensional cubic box (10nm×10nm×10nm). Essentially, the three-dimensional Cartesian coordinates are randomly assigned within the simulation box to the center of mass of the first protein. A random orientation the along three-dimensional Euler angles is then randomly generated to rotate the molecule, which is centered at its newly assigned center of mass. The same procedure is followed for the second protein. This newly generated random conformation will be further checked to exclude any steric clashes between two binding partners. Moreover, if multiple trajectories are carried out, the initial conformation in each individual trajectory is randomly regenerated so that there is no correlation between any two trajectories. The construction of random initial orientations between two proteins which are different from multiple trajectories reflects the realistic conditions in experimental systems and can prevent the simulations from attaining non-physical biases in transition states. After the simulation starts, both binding partners undergo random translational and rotational diffusions within each simulation step. The translational and rotational diffusion constants were obtained by fitting data calculated by a precise boundary element model [66,67]. This hydrodynamic model utilizes the excluded volume of a rigid protein that is obtained from crystallographic coordinates and surrounds the protein with a uniform hydration thickness. It has been found to yield properties such as diffusion coefficients in excellent agreement with the experiment and is more than 10 times as successful as traditional bead-based methods. Diffusions are further guided by the intermolecular interactions, which are described by a hybrid force field introduced in the next section. A periodic boundary condition is then applied to any protein that reaches the boundary of the simulation box.

A new configuration for different binding partners in the simulation box is obtained after each step of diffusions. The total energy of intermolecular interactions is derived for the new configuration, based on the hybrid force field. The probability of accepting the diffusions is further calculated based on the metropolis criterion, by comparing the total energy of the old configuration before diffusions and the new configuration after diffusion [68]. If the new configuration is accepted, we further check whether an encounter complex can be formed between two binding partners based on a predefined association criterion. Based on our previous study, we assume that an encounter complex is formed if at least three native-like contacts between two binding partners are restored. A native-like contact between the functional centers of two residues is restored if the difference in their distance is less than 2 Å from the original distance observed in the native complex. In the current study, we further explored the combination of other different criteria, such as the percentage of restored native contacts, the root-mean-square difference (RMSD) between the current configuration and the native structure of the protein complex, and the distance between the interfaces of two binding partners. Detailed results obtained using the combination of different association criteria are discussed in the results. Finally, if an encounter complex is formed, the current simulation trajectory will be terminated. Otherwise, the simulation continues until it reaches the maximal time duration (Figure 1). In this study, each simulation trajectory consists of 10^3^ steps, and each time step is 1ns, so that the total simulation time for each trajectory is 1μs.

In order to calibrate the computational performance of the method, we also use the test system 2VLN as a specific example. It takes approximately 10 seconds to generate a trajectory of 100 ns on a regular Linux desktop. Based on previous literature, it has been shown that for a protein complex with normal size, it takes on average one hour to generate a typical 100 ns trajectory of a Brownian dynamic (BD) simulation on a regular Linux desktop [69,70]. This indicates that our coarse-grained (CG) simulations are much faster than the traditional all-atom BD simulations. Finally, this KMC simulation program is now available for download at https://github.com/wulab-github/KassKMC. This package contains all the source codes used for simulation in the FORTRAN77 programming language and as an executable file. The parameter files which contain all the force constants in the statistical potential are included. The package also contains the list of 62 protein complexes in the benchmark set. The package further offers a demonstration example (PDB 2VLN) with both the protein structure in PDB format as an input file and an output file that contains the simulation results of 10^4^ trajectories. The program work on a Linux platform and downloading is free for academic users.

### 2.3. A Hybrid Force Field of Intermolecular Interactions for Guiding Monte Carlo Simulations

We constructed a hybrid force field to describe the total intermolecular energy between two binding partners of a protein complex. As mentioned in the last section, this force field is used to guide the Monte Carlo simulations of protein complex association. It can be written as follows:

(1) $$E_{tot}=\left(1-w\right)\times E_{physics}+w\times E_{statistics}$$

Equation (1) consists of two terms: a physics-based potential and a statistics-based potential (Figure 1). The parameter ω defines the relative contributions of these two potentials. The physics-based potential can further be written as follows:

(2) $$E_{physics}=\sum_{i,j}q_{i}q_{j}/\left(4\pi \varepsilon_{0}D_{eff}r_{ij}\right)+\omega_{\alpha }\sum_{i,j}\left[HP_{i}+HP_{j}\right]+\sum_{i,j}\varepsilon_{ij}\left[{\left(\frac{\sigma_{0}}{r_{ij}}\right)}^{12}-{\left(\frac{\sigma_{0}}{r_{ij}}\right)}^{6}\right]$$

The first term is the Kim–Hummer model [71,72], which describes the electrostatic interaction between the *i^th^* and the *j^th^* residues at the binding interface of a protein complex, where *q_i_* gives the charge of residue *i* at the functional center of its side-chain, and *ε_0_* is the vacuum electric permittivity. In order to capture the shielding effect between two residues, an effective dielectric coefficient, $D_{eff}=D_{s}\exp\left(r_{ij}/\xi \right)$, was defined in which *ξ* is the Coulomb Debye length used to mimic the screening effect at different ion strengths. The second term of the physics-based potential is used to estimate the hydrophobic effect between proteins, in which *HP_i_* is the Kyte–Doolittle hydrophobic score for residue *i* with its corresponding type [73]. The weight constant *w_α_* is used to balance the hydrophobic and electrostatic interactions. Finally, the third term takes into account the excluded volume effect. The depth of the potentials *ε_ij_* is a step function depending on the distance between two specific representative sites *r_ij_*. It equals 0 if the distance is larger than *σ_0_*; otherwise, it equals 5kT. The parameter *σ_0_* has constant values which depend on the type of distance between the two representative sites. In particular, like the values used in our previous study, it equals 3.8 Å between two Cα atoms, 2.8 Å between a Cα atom and a side-chain functional center and 2.2 Å between two side-chain functional centers, respectively.

In addition to the physics-based potential used in our original simulation method, we further introduced a statistics-based potential that was derived from analyzing the available protein complexes in the current structural database [74]. In detail, it has the following form:

(3) $$E_{statistics}=\sum_{ij\in \mathrm{int}er}g\left\{\left|u_{SC}\left(i,j\right)\right|\times \left[{\left(\frac{r_{ij}^{0}}{r_{ij}}\right)}^{12}-f\left(u_{SC}\left(i,j\right)\right)\times {\left(\frac{r_{ij}^{0}}{r_{ij}}\right)}^{6}\right]\right\}$$

The summation in Equation (3) is taken across all residue pairs at the interface between two binding partners of a protein complex, which is recognized if the inter-molecular distance between any atoms of their side-chains is less than 5.5 Angstrom. The distance between the functional centers of these residues is $r_{ij}$, while $r_{ij}^{0}$ gives their relative distance in the native structure. The function *f(x)* equals 1 when x is smaller than 0 and -1 when x is larger than 0. This function ensures that the negative energy parameters derived from the statistics are attractive, while the positive parameters are repulsive. On the other hand, the function *g(x)* equals -1 when x is smaller than 0 and 0 when x is larger than 0. In turn, *u_sc_(i,j)* determines the strength of the interaction between residue types *i* and *j*. The specific value of the parameter was derived from the statistics of structurally available protein complexes [75]. In detail, it can be written as follows:

(4) $$u_{sc}(i,j)=-kT\ln\frac{N_{obs}(i,j)}{\chi_{i}\chi_{i}N_{obs}}$$

In Equation (4), $N_{obs}\left(i,j\right)$ is the observed number of pairs between residue type *i* and type *j*, which form contact at the binding interfaces; the denominator in the equation represents the expectation number of pairs between residue type *i* and type *j* under the quasi-chemical approximation [76,77], in which $N_{obs}$ represents the total residue pairs at the binding interface and *χ_i_* represents the mole fraction of residues with type *i* at the binding interfaces. Two residues are considered to form a contact if a pair of any atoms belonging to the side-chains of these residues is closer than the distance cut-off value (5.5 Angstrom). In order to obtain different values for the energy parameter, we counted all the observed numbers of corresponding residue pairs in Equation (4) through a large-scale structural library of protein complexes that was constructed based on the original 3did database [75]. The database selected all inter-domain interactions from protein complexes for which high-resolution three-dimensional structures are available. We further reduced the sequence redundancy from this database, which led to a final library consisting of 4960 entries of protein–protein interactions. The detailed procedure of extracting the energy parameters was described in our previous study [74].

Finally, it is worth mentioning that in our simulation of protein–protein association, we only consider the intermolecular non-covalent interactions between two binding partners. We assume that the tertiary structure of each individual protein is well conserved during the process of association. In order to reach this goal, all the intramolecular degrees of freedom are fixed, and proteins undergo translational and rotational diffusions as rigid bodies in the simulation. As a result, it is not necessary to reflect the covalent bonds that are used to maintain protein tertiary structures in our model.

## 3. Results

In order to systematically test the generality of our kinetic Monte Carlo (kMC) simulation in estimating the association rates of different protein complexes, we collected 62 protein complexes from previous literature as a benchmark set. The criteria of benchmark construction are described in the Methods. Detailed information about the benchmark set, including the PDB IDs, the index of two binding partners and their corresponding experimental values of association rates, is listed in Table S1 in the Supplementary Materials. For all these protein complexes, 10^4^ trajectories were carried out by the kinetic Monte Carlo simulation algorithm (a detailed description of the algorithm can be found in the Methods and in Figure 1). Each trajectory started from a different initial random configuration. After the initial conformation, diffusions of each binding are guided by the intermolecular energies. At the end of each trajectory, two binding partners either successfully or unsuccessfully form an encounter complex through the pre-defined association criteria. Based on counting how many encounter complexes are observed from all the trajectories, the association probability can be derived for each complex in the benchmark set. The calculated association probability *P_i_* for complex *i* will be further converted into a rate constant $k_{on}^{i}$ by the following equation:

(5) $$k_{on}^{i}=k_{on}^{\min}\times 10^{\left(\frac{P_{i}-P_{\min}}{P_{\max}-P_{\min}}\right)\times N}$$

The parameters *P_min_* and *P_max_* in Equation (5) stand for the minimal and maximal values of association probabilities that were obtained from all the protein complexes in the benchmark. The parameter $k_{on}^{\min}$ equals 1.03 × 10^5^, which stands for the lowest value of experimentally measured association rates observed in the benchmark. The parameter *N* indicates how many orders of magnitudes for experimental association rates are considered in our benchmark. As described in the Methods, because we only considered protein complexes whose rate constants are within the range of 1.0 × 10^5^ and 1.0 × 10^9^ M^−1^s^−1^, the value of *N* equals 4 in Equation (5).

The force field which was developed to delineate the intermolecular interaction in our kinetic Monte Carlo simulations combines a previously constructed physics-based potential with a newly added statistics-based potential. A weight constant, ω, is used to define the relative contributions of these two potentials, as shown in Equation (1). In order to explore the capability of this new hybrid force field in estimating the association rates of different protein complexes, we adjusted the value of this weight constant from 0 to 1. Only the physics-based potential is used in the simulations when the weight constant equals 0, while only the statistics-based potential is used when the weight equals 1. Given a specific value for the weight constant, the association rate was calculated for each protein complex in the benchmark based on the statistical analysis from its 10^4^ simulation trajectories, as illustrated in the previous paragraph. We compared the calculated association rates of all protein complexes with their corresponding experimental measurements. Consequently, the Pearson correlation coefficient (PCC) between these two datasets was obtained under different values of weight constant.

**Figure 2:**
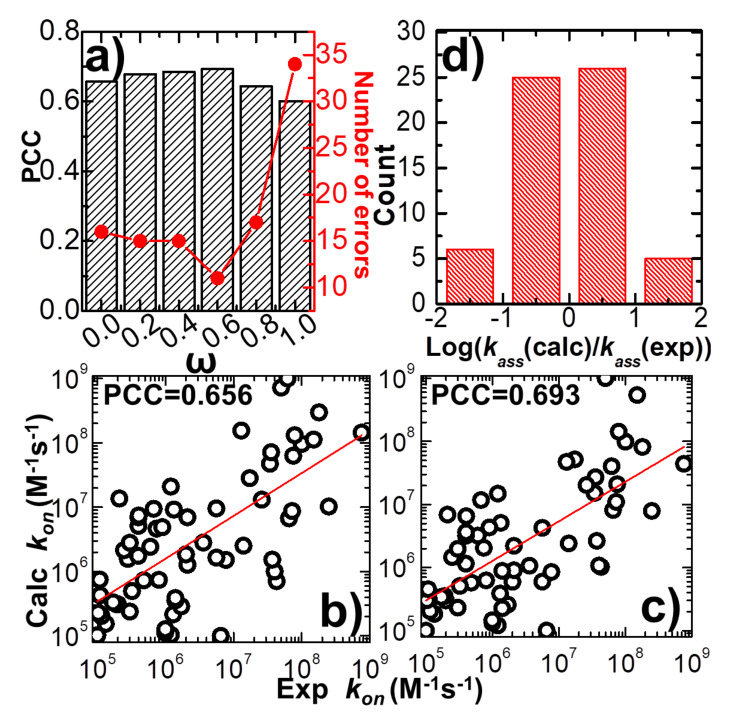
We adjusted the value of parameter ω, which balances the weights between the physics-based and statistics-based potentials from 0 to 1. Under each specific value, the association rates were calculated for all protein complexes in the benchmark and compared with their corresponding experimental measurements. The calculated Pearson correlation coefficient (black bars) and the number of errors (red dots and line) in the correlation between simulated and experimental association rates are plotted in (**a**) as a function of the value of this weight constant. One-to-one detailed comparisons between simulated and experimental association rates are shown in the log 10 base plots when the value of the weight constant equals 0.0 (**b**) and 0.6 (**c**), respectively. The red lines are from the linear regression fit between simulated and observed log_10_*k_on_* values. The slope of the red line in (**b**) equals 0.66 (standard error equals 0.093) and the intercept equals 2.21 (standard error equals 0.61), while the slope of the red line in (**c**) equals 0.63 (standard error equals 0.084) and the intercept equals 2.35 (standard error equals 0.55). We further calculated the common logarithm of the ratio between simulated and experimental association rates. The distribution of our calculations for all 62 protein complexes is plotted on histograms in (**d**).

In detail, the variation between weight constant and calculated PCC is plotted in Figure 2a. The figure shows that without the statistics-based potential (ω = 0), we can still achieve a positive correlation between simulations and experiments. A log 10 base plot between our calculated values of association rates and their experimental data for all 62 complexes is displayed in Figure 2b under this circumstance, with a Pearson correlation coefficient of 0.656. When the statistics-based potential started to be added into the force field, Figure 2a suggests that a higher correlation was obtained. The value of PCC peaks when the weight constant equals 0.6. The comparison between simulated and experimental association rates for all 62 complexes is displayed in Figure 2c under this circumstance, with a Pearson correlation coefficient of 0.693. However, the correlation between simulations and experiments will drop if the weight of the statistics-based potential increases further. Finally, the lowest correlation (PCC = 0.6) was attained without the physics-based potential (ω = 1). Taken together, our simulation results indicate that our kinetic Monte Carlo simulation can distinguish the kinetics of protein–protein interactions within a wide range of association rates. Moreover, the optimal combination between the physics-based and the statistics-based potentials was able to improve the simulation’s accuracy.

The common logarithm of the ratio between simulated and experimental association rates $\log_{10}(k_{on}^{calc}/k_{on}^{\exp})$ was further calculated for all complexes in the benchmark, under the optimal value of weight constant (ω = 0.6). If this value was larger than 1 for a protein complex, its association rate was overestimated over an order of magnitude relative to the experimental data. On the other hand, a protein complex with a value smaller than -1 indicates that its association rate was underestimated over an order of magnitude relative to the experimental data. Figure 2d shows the distribution of our calculations. The histogram in the figure indicates that among all 62 complexes, only six were underestimated over an order of magnitude, and five were overestimated over an order of magnitude. The association rates of the remaining 51 protein complexes were reproduced within one order of magnitude. We assume that the association rate of a protein complex can be correctly predicted if the difference between our calculated value and its corresponding experimental data is below one order of magnitude. This is a reasonable estimation given the fact that the experimentally observed association rates span an extremely wide range, with over ten orders of magnitudes. Based on this criterion, we show that our simulation to predict the protein–protein association rates can reach an overall accuracy of 80%. The calculated association probabilities and rates for all protein complexes in the benchmark under the weight constant 0.6 are listed in Table S1.

Based on the definition of $\log_{10}(k_{on}^{calc}/k_{on}^{\exp})$, we further counted the number of errors in the prediction of association rates for all protein complexes in the benchmark. The error of prediction for a specific protein complex is marked as its absolute value of calculated $\log_{10}(k_{on}^{calc}/k_{on}^{\exp})$, which is larger than 1, indicating that the difference between experimental and predicted association rates is higher than one order of magnitude. The total numbers of errors from the prediction were derived under all different values of the weight constant ω. The values are plotted in Figure 2a as red dots and lines. The figure shows that the change in prediction errors as a function of weight constant ω correlates well to the variation in PCC. The highest value of PCC corresponds to the lowest number of errors when ω equals 0.6. This result confirms that the accuracy in predicting the rates of protein–protein association can be marginally improved when an original version of the physics-based potential is complemented with a new statistics-based potential with an optimal weight.

**Figure 3:**
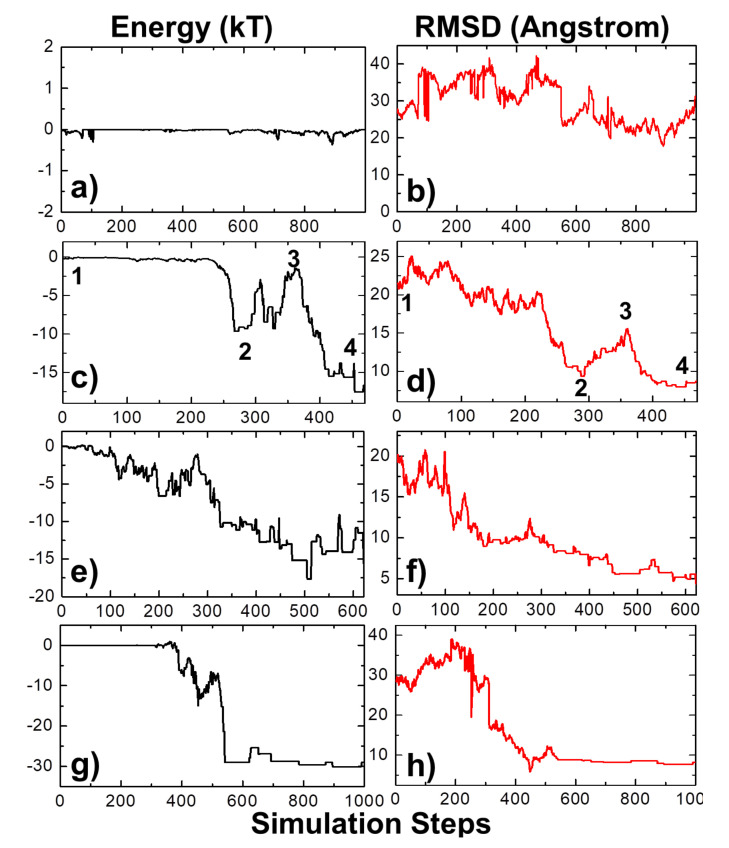
The protein complex 2VLN was selected from the benchmark as a specific example to characterize the dynamic mechanisms of association. Among its 10^4^ simulation trajectories, we further picked four representatives to illustrate the detailed association processes. The changes in the total intermolecular interactions between two binding partners in these four trajectories are plotted by black curves on the left side in (**a**), (**c**), (**e**) and (**f**) as a function of simulation steps, while their changes in the root-mean-square difference (RMSD) from the native complex are plotted on the right side by red curves in (**b**), (**d**), (**f**) and (**g**), respectively. Finally, four representative kinetic steps along the association pathways are marked by the numbers “1”, “2”, “3” and “4” in (**c**) and (**d**).

We selected a protein complex from the benchmark as a specific example to characterize the dynamic mechanisms of association. In detail, the complex formed between the E9 DNase domain of colicin endonucleases and immunity protein Im9 (PDB id 2VLN) was selected as a test system [78]. Among a total number of 10^4^ simulation trajectories, with a maximal duration of 1000 ns for each individual trajectory, we found that encounter complexes were successfully formed in 1819 of them. This indicates that the value of the association probability equals 0.1819. Using Equation (5), we further calculated the rate of association between proteins E9 DNase domain and Im9. The association rate of the complex equals 9.86 × 10^7^ M^−1^s^−1^, which is close to the experimental measurement (1.0 × 10^8^ M^−1^s^−1^) [78]. The physical processes along four representative simulation trajectories are illustrated in Figure 3. The changes in the total intermolecular interactions between two binding partners in these four trajectories are plotted by black curves in Figure 3a,c,e,f as a function of simulation steps, while their changes in the root-mean-square difference (RMSD) from the native complex are plotted by red curves in Figure 3b,d,f,g, respectively. The RMSD is large at the beginning of all four trajectories, resulting from their initial random configurations. As a result, the intermolecular interactions at the initial stage of association in all four systems are very close to 0, indicating that two binding partners were separated from each other and diffused independently in the simulation box.

**Figure 4:**
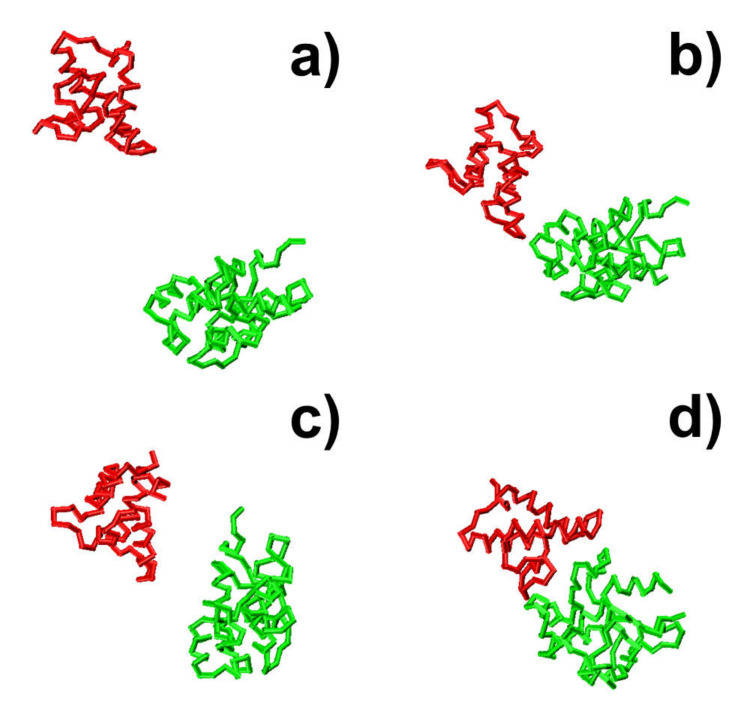
The snapshots of four representative kinetic steps along the association pathways are illustrated for protein complex 2VLN. Two binding partners in the complex are shown in red and green, respectively. The initial configuration is shown in (**a**). In (**b**), a tentative binding interface is formed between two binding partners, corresponding to a local energy minimal state denoted by the number “2” in Figure 3. This tentative complex was then dissociated, and two binding partners were reorganized into a different orientation, as illustrated in (**c**) and the number “3” in Figure 3. At the end of the simulation, the final encounter complex forms, as shown in (**d**).

In the first system, small fluctuations in total energy were observed at around 0 throughout the simulation trajectory, as shown in Figure 3a. In comparison, a high level of RMSD was sustained along the trajectory, with large fluctuations (Figure 3b), indicating that the proteins cannot associate into complexes by the end of the maximal time duration in this system. On the contrary, complexes were successfully formed in the next three trajectories. This is reflected by the fact that the intermolecular interactions dropped to negative values in these systems, while their RMSD values also decreased to levels below 10 Å by the end of the simulations. In these cases, the proteins have spatially approached each other and found their actual binding sites after they diffused throughout the simulation box. Additionally, Figure 3 shows that the formation of the protein complex is faster in some trajectories than others. For instance, the complex in the second trajectory was formed at 470 ns, whereas the complex in the third trajectory was formed at 630 ns. Moreover, in most of these trajectories, we found that local energy minimal states were formed between proteins along the pathways to their final associations. Similar to the process of folding [79], protein complexes are able to overcome the energy barrels between these local minimal states and reach the final native-like configuration. For instance, in the second trajectory, following the initial configuration, which has an energy level of zero and a large RMSD value, we found that the system formed a relatively stable structure, in which both energy and RMSD dropped to relatively low levels. The initial configuration is denoted by the number “1” in Figure 3c,d, and its corresponding snapshot from the trajectory is shown in Figure 4a. The local energy minimal state is denoted by the number “2” in the figures, and its corresponding snapshot from the trajectory is shown in Figure 4b. A tentative binding interface is formed between two proteins, as observed in the figure. After the formation of this tentative complex, both the energy in the system and the RMSD of the protein complex started to increase and peaked at the point denoted by the number “3” in Figure 3c,d and the corresponding snapshot shown in Figure 4c. The comparison between structures in Figure 4c suggests that the tentative complex was dissociated, and two proteins were reorganized into a different orientation. Finally, both the energy in the system and the RMSD of the two proteins started to drop again until they formed the final encounter complex, which is denoted by the number “4” in Figure 3c,d and the corresponding structure shown in Figure 4d. The comparison between structures in Figure 4b,d suggests that a more compact complex was formed in the final configuration compared to the tentative complex.

Taken together, the analysis of simulation details reveals the dynamic mechanism of association. We showed that the individual association pathway is a multistep process which involves continuously searching local binding configurations and repeatedly forming kinetic intermediates with dissociation and re-association. A similar phenomenon has also been observed in a previous study using all-atom molecular dynamic simulations [33,34].

**Figure 5:**
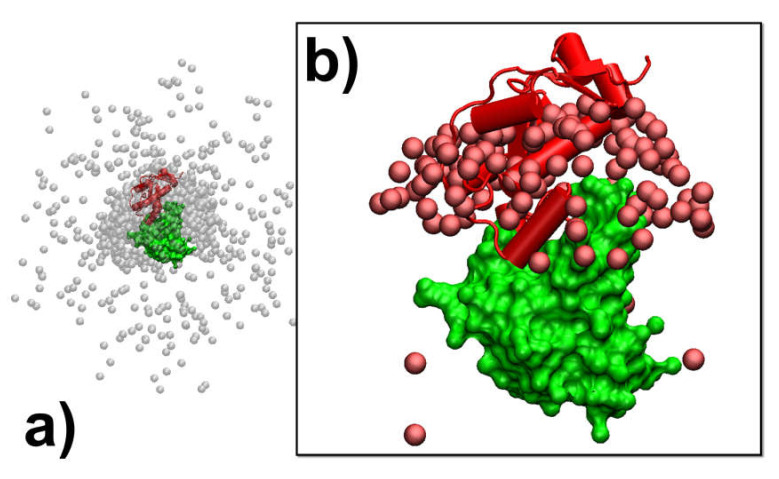
We plotted the spatial distribution of 10^3^ randomly selected final configurations from all the 10^4^ simulation trajectories of protein complex 2VLN. In (**a**), the native structure of the protein complex is placed in the center of the box, while the protein E9 DNase domain is shown in green with its surface profile and Im9 is shown in red in the cartoon representation. The structures of protein E9 DNase domain from our simulations were aligned with the native configuration, while the centers of mass for their corresponding protein Im9 are represented by the grey points. In (**b**), we further plotted the spatial distribution of those configurations in which encounter complexes were successfully formed. The centers of mass for the protein Im9 in these configurations are represented by the red points.

In addition to the analysis of the individual association pathways, it is also helpful to compare results between different simulation trajectories. As a result, protein complex 2VLN was still used as a test system. We plotted the spatial distribution of the final configurations that were generated from all the simulation trajectories in order to obtain an overview of the structural similarity among all the successfully formed encounter complexes. This distribution is plotted in Figure 5a. In the figure, the native structure of the protein complex is placed in the center of the box, while the protein E9 DNase domain is shown in green with its surface profile and Im9 is shown in red in the cartoon representation. In order to attain a better visual effect, 10^3^ final configurations were randomly selected from the 10^4^ trajectories to avoid spatial crowding in the figure. The structures of protein E9 DNase domain in these configurations were aligned with the native configuration, while the centers of mass for the protein Im9 are represented by the grey points. Figure 5a shows that there are a number of grey points uniformly distributed in the simulation box. These points represent the situation in which protein Im9 still diffuses around its binding partner. With the exception of these configurations, the majority of grey points are distributed around the center. These points represent the situation in which Im9 has formed a physical interface with protein E9 DNase domain.

According to the association probability calculated for protein complex 2VLN, encounter complexes were successfully formed in about 200 final configurations out of 10^3^ selected trajectories. We further plotted the spatial distribution of these configurations in Figure 5b. As in Figure 5a, the native structure of the protein complex is placed in the center of the box, with the protein E9 DNase domain in a green surface profile and Im9 in a red cartoon representation, while the centers of mass for the protein Im9 are represented by the red points. The figure shows that the centers of mass for almost all Im9 proteins in the encounter complexes are distributed relatively closer to the native conformation than the distribution observed in Figure 5a, although a highly noticeable range of spatial deviation exists. This indicates that encounter complexes constitute an ensemble of loosely bound structures formed around their native conformation. This ensemble of encounter complex thus suggests that the transition states of protein–protein association could be highly diverse on the structural level. Furthermore, the combination of Figure 5a,b with the previous two figures enables the following mechanistic insights into the physical process of protein association. There are overall three outcomes observed in the simulations of association. In particular, two binding partners either 1) freely diffuse by the end of the maximal time duration, 2) form a physical interface that is different from the native conformation and remain kinetically trapped in this nonnative state, or 3) form an initial tentative contact, that is different from the native conformation, but later successfully transit into the correct binding interface through the process of dissociation and re-association. Moreover, for all trajectories in which encounter complexes were formed through the third pathway, their structures displayed a wide variety of relative protein–protein orientations around the native conformation.

In summary, the analysis of the overall simulation trajectories reveals, firstly, that association between proteins could be kinetically trapped into nonnative states, secondly, that the pathway towards the final formation of the encounter complex is a dynamic process consisting of local structural reorganization and, finally, that even the structures in the final ensemble of encounter complexes are highly diverse. These features are consistent with the observations in another previous study using all-atom molecular dynamic simulations [33,34].

**Figure 6:**
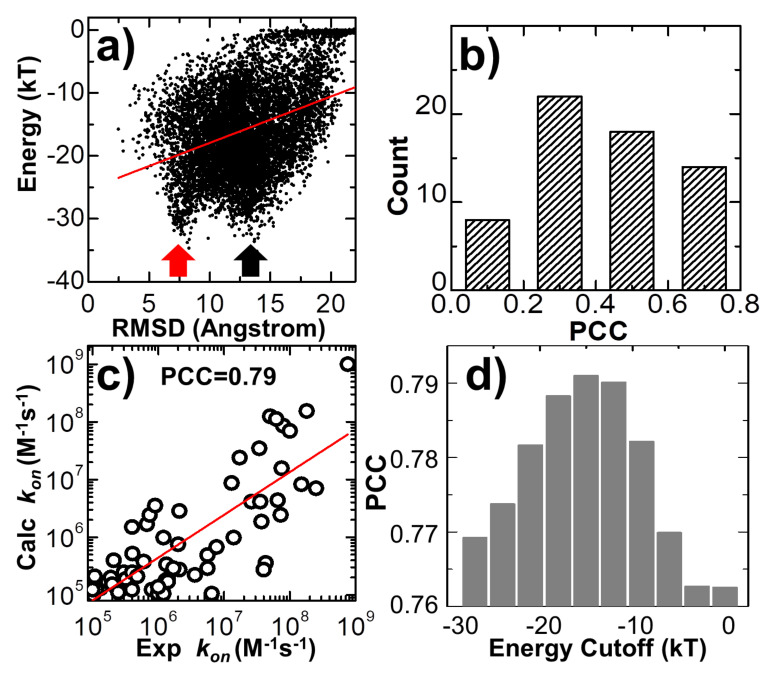
We compared the values of RMSD with intermolecular energy for all the 10^4^ final configurations taken from the simulations of protein complex 2VLN in (**a**). We also calculated the correlation between RMSD and intermolecular energy for all 62 protein complexes in the benchmark. The distribution of these calculated Pearson correlation coefficients is plotted in (**b**). We further enumerated the combination of different binding criteria for encounter complex formation. The log 10 base plot in (**c**) shows that by optimizing the combinations and values of different binding criteria, we were able to achieve a high correlation between experimental and simulated association rates for all protein complexes in the benchmark. Finally, using energy cutoff as an example, we found that the high correlation between simulation results and experimental data could only be achieved when the values of the binding criteria were adopted within a small range (**d**).

In the last three trajectories plotted in Figure 3, we found that the decreases in the intermolecular energy in the systems co-variated with the RMSD between the conformation of the protein complex and its native structure. This suggests that the simulations of association were driven by the intermolecular energy. In order to further test whether this energetic variable could be used as an indicator to capture the features shared among encounter complexes, we compared the values of RMSD with intermolecular energy for all the final configurations taken from the end of 10^4^ trajectories. The correlation is shown in Figure 6a for the complex 2VLN. Each of the ten thousand points in the figure stands for the final configuration from one trajectory, while its *y*-axis equals the intermolecular energy calculated based on this configuration, and the *x*-axis is its RMSD from the native structure of the protein complex. The group of trajectories in which two binding partners failed to form a contact are reflected by the tip in the top-right corner of the figure. The distribution below the tip, on the other hand, indicates a large variety of complexes formed between these two binding partners in the remaining trajectories. We found a strong positive correlation in this distribution. The calculated Pearson correlation coefficient equals 0.7. In particular, a large group of encounter complexes formed through the lowest intermolecular energy, as indicated by the red arrow in Figure 6a, show small values of RMSD of around 7 Å. We further calculated the Pearson correlation coefficient between RMSD and intermolecular energy from the end of the 10^4^ simulation trajectories for all 62 protein complexes in the benchmark. The distribution of Pearson correlation coefficient values is plotted in Figure 6b. The figure shows that the correlations for all protein complexes are positive, while more than half of them have strong correlations that are higher than 0.5. This positive correlation between binding energy and RMSD from the native structures suggests that protein complexes formed with native-like protein interfaces tend to have lower binding energies than complexes that are trapped in the non-native states. As a result, the structural similarity embedded in the ensemble of encounter complexes can be characterized by the intermolecular energy. In other words, the positive correlation between energetic and structural features in protein complex formation indicates that protein–protein association is a kinetic process similar to protein folding [79], in which diffusions of binding partners are driven by a “funnel-like” energy landscape.

In the last section of the Results, we explored the combination of different criteria for encounter complex formation. As described in the Methods, the number of intermolecular contacts recovered from the native structure was used as a single criterion to determine whether an encounter complex can be successfully formed along simulations. Here, in addition to the number of native contacts, we further considered four other criteria. The first criterion is that the percentage of native contacts must be above a cutoff value. The percentage is calculated by the ratio of the number of recovered native contacts to the total number of intermolecular contacts in the native complex. The second criterion is that the RMSD between the encounter complex and the native complex needs to be below a cutoff value. The third is the distance cutoff between the interfaces of two binding partners, while the last criterion is the energy cutoff calculated by Equation (1). The number of encounter complexes observed in all simulation trajectories strongly depends on the combinations and values of these different binding criteria. We enumerated all different combinations for these criteria. For each combination, a wide range of discretized values was adopted for different criteria. In detail, the range of native contact numbers was initially 0 to 10, with an interval of 1; the range of the native contact percentage was initially 0% to 10%, with an interval of 1%; the range of RMSD was initially 0 to 30Å, with an interval of 3Å; the range of interface distance cutoff was initially 0 to 10Å, with an interval of 1Å; and the range of binding energy cutoff was initially 0 to -30kT, with an interval of 3kT. For all possible combinations and values, we calculated association probabilities for each protein complex in the benchmark. Consequently, we found that by selecting the optimal combinations and values of these binding criteria, we were able to achieve a higher correlation between experimental and simulated association rates than using individual binding criterion. One example is shown in Figure 6c. The specific combination of binding criteria adopted in the figure is as follows: the native contact percentage should be higher than 6%; the native contact number should be more than 5; the binding energy should be lower than -15kT; finally, the RMSD and interface distance should be lower than 12 and 10 Å, respectively. Under this combination of binding criteria, a high PCC value of 0.79 was achieved.

We further found that this correlation also depends on the values of the adopted binding criteria. For instance, in Figure 6d, we found that a high correlation between simulation results and experimental data can only be attained when the value of energy cutoff was adopted within a small range. For instance, the best PCC of 0.79 could only be attained with an energy cutoff of -15.0kT, as shown at the end of the last paragraph. Under higher energy cutoff values, e.g., -3.0kT, the highest PCC found in the system is 0.76, if the native contact percentage is higher than 4% or the native contact number is more than 5, with the RMSD and interface distance cutoffs equal 15 and 1 Å, respectively. Similarly, under lower energy cutoff values, e.g., -27.0kT, the highest PCC found in the test is also lower than 0.77, if the native contact percentage is higher than 3%, or the native contact number is more than five, with the RMSD and interface distance cutoffs equal to 12 and 10 Å, respectively. This can be explained as follows: if the energy cutoff is too low, the criterion for association will not be strict enough and systems will contain encounter complexes that are not correctly bound. As a result, the association probability will be overestimated. On the other hand, if the energy cutoff is too high, the criterion for association will be too strict and systems will not contain enough encounter complexes that are correctly bound. As a result, the association probability will be underestimated. In general, the range of energy cutoff that results in a high correlation with experimental association rates corresponds to the scope of average binding energy that is found in the transition state ensemble of all protein complexes. Taken together, our statistical results imply that although there is strong diversity in the structures and energetics of different protein complexes, the common mechanisms underlying their association can be characterized by a combination of different binding criteria.

## 4. Concluding and Discussions

The versatile functions of protein complexes in cells strongly depend on how quickly the building blocks in these complexes can be assembled together [80,81,82]. This kinetic property of protein–protein interactions is quantified by the association rate, which can be traditionally measured through various experimental methods. Compared with these methods, computational modeling approaches are much less time-consuming and labor-intensive. Moreover, they can provide a mechanistic understanding of biophysical problems on a spatial-temporal level, which is currently inaccessible in the laboratory. Relative to other all-atom simulation techniques that are highly demanding in terms of computational resources, we study the physics process of protein–protein association using a coarse-grained model and a new hybrid force field which contains both physics-based and statistics-based potentials. Our physics-based potential consists of two terms that describe the electrostatic interactions and hydrophobic effect. The parameter used to balance the weights between these two factors has been tuned in order to achieve the best performance, as in our previous study [62]. However, there are various types of molecular interactions missing in this simplified potential, such as the side-chain hydrogen bonds, the dipole–dipole interactions and the π-stacking of aromatic rings between different side-chains. We assume that the contributions of these complicated energy contributions can be implicitly included in the statistics-based potential, thereby complementing the original physics-based potential. This assumption was verified by our results, in which we showed that a mixed form of these two different potential functions can improve the correlation between simulated and experimental association rates, although this improvement is not substantial. The best correlation was obtained when the weight constant between the contributions of the physics-based and statistics-based potentials was equal to 0.6. However, it is worth mentioning that the value of this weight depends on the protein complexes tested in the benchmark. In other words, when a various selection or a subset of benchmarks is used, it is likely that the best correlation will be derived under a different value of weight constant. Nevertheless, it is beyond the scope of this study to offer an optimal value of weight constant that can provide the best prediction of the association rate of any protein complex. The purpose here is to explore the idea that a mixture of force fields derived from complementary sources is better able to describe the process of protein–protein association.

Previous works showed that the association rate constant of forming transient complexes purely via unbiased diffusions in which the diffusion coefficients of individual proteins can be calculated by methods such as Hydropro [83] is ~10^5^M^−1^s^−1^ [50,84,85]. The real values of association rates higher than this “basal” rate constant are the result of the intermolecular interactions between proteins, such as the long-range electrostatic attraction, which biases diffusions toward the formation of transient complexes. As a result, the association rates calculated from simulation models that incorporate both diffusions and intermolecular interactions, such as the method developed in this study, can differ by several orders of magnitude and thus are closer to the experimentally observed values. We systematically calibrated our simulation method against a large-scale benchmark set. For each complex in the benchmark, a large number of simulation trajectories were carried out. Based on the statistical analysis of these trajectories, we found that common mechanisms underlie the association of structurally diverse protein complexes. In particular, we revealed that the association of a protein complex contains multiple steps, in which proteins continuously search their local binding orientations and form non-native-like intermediates through repeated dissociation and re-association. Moreover, we showed that encounter complexes constitute an ensemble of loosely bound structures formed around their native conformation, suggesting that the transition states of protein–protein association could be highly diverse on the structural level. Finally, a positive correlation between the binding energy and structural similarity of encounter complexes with their native conformation was found, which indicates that protein–protein association is a kinetic process in which the diffusions of binding partners are driven by a “funnel-like” energy landscape. We also noticed that encounter complexes formed in a number of trajectories have a low binding energy but a large RMSD from the native structure, as shown by the black arrow in Figure 6a, indicating that the binding partners of the protein complexes in these trajectories associate into non-native-like encounter complexes. These low-energy, non-native-like encounter complexes cannot be discerned by other binding criteria either, including the number or the percentage of native contacts and the distance between the binding interfaces. The formation of these complexes is either due to the fact that they are kinetically trapped in the intermediate states during the repeated dissociation and re-association process along their association pathways or due to the large energy frustration in the simplified force field used in this study. In summary, these results shed light on our understanding of how protein–protein recognition is kinetically modulated, and the computational method developed in this study can therefore complement existing experimental approaches that measure protein–protein association rates.

In our previous study, the association rates were derived directly from the calculated association probability and related simulation parameters, such as volume and length of association time. However, the potential used to describe the intermolecular interactions in the simulation is simplified and thus not accurate enough, so the energy landscape for guiding association is not funnel-like. As a result, one problem in estimating the association rates in our previous study is that the association with high experimental rate constants tends to be underestimated, while the association with low experimental rate constants tends to be overestimated. To tackle this issue, in this study, we attempt to use an empirical formula (as shown in Equation (5)) to minimize the effect of the simplification of force field. It is worth mentioning that this equation is only applied to rescaled simulation results. There is no free parameter which can be adjusted to fit against the experimental data. The parameters $k_{on}^{\min}$ and *N* are experimental constants observed in the benchmark. The association probabilities *P_i_* were derived from the first-principle simulation, while their upper and lower limits *P_min_* and *P_max_* were fixed variables, as long as all the association probabilities were derived. Therefore, there is no issue of overfitting when using Equation (5) to compare our simulation results with experimental data. However, it is possible that this empirical formula could bring some artifacts. For instance, the probability of association is time dependent. It will approach a value of 1 when given an extremely long simulation time, but it will approach 0 with an extremely short simulation time. This effect, fortunately, can be potentially canceled out by calculating the relative difference of association probability from its maximal and minimal values, as shown in the equation. Moreover, the length of simulation duration in our study is carefully determined to obtain an appropriate range of association probability, while the same value was applied to all protein complexes in the benchmark. Additionally, there is another limitation in Equation (5). It only takes into account the association rates across four orders of magnitude, as the range considered in the benchmark set. Although we adopted this limited range of rate constant values in the equation, it can still be performed to study protein associations whose rates are beyond this range. For instance, if the simulated probability for one protein complex is smaller than the minimal probability in Equation (5), this will lead to the result that the calculated association rate is lower than 10^5^M^−1^s^−1^. On the other hand, if the simulated probability for one protein complex is larger than the maximal probability in Equation (5), this will lead to the result that the calculated association rate is higher than 10^9^M^−1^s^−1^.

## Supplementary Materials

The following are available online at https://www.mdpi.com/2218-273X/10/7/1056/s1, Table S1: the list of all protein complexes in the benchmark.
