# Supplementary material for: Using Coarse-Grained Simulations to Characterize the Mechanisms of Protein–Protein Association
Source: Biomolecules. 2020 Jul 15;10(7):1056. doi: 10.3390/biom10071056 (PMC7407674; doi:10.3390/biom10071056)
Supplement: Supplementary file 1 [file biomolecules-10-01056-s001.pdf]

PDB	Binding Partner 1	Binding Partner 2	Ionic Strength (M)	Exp k_{on} ($M^{-1}s^{-1}$)	$Prob_{Ass}$	Calc k_{on} ($M^{-1}s^{-1}$)
1GL0	E	I	0.1	103000.0	0.0324	3.03E5
3N06	A	B	0.1	110000.0	0.0334	3.15E5
1MQ8	A	B	0.16	133000.0	0.0195	1.84E5
1CBW	FGH	I	0.1	170000.0	0.0355	3.42E5
1FC2	C	D	0.1	190000.0	0.0363	3.52E5
2I26	N	L	0.1	210000.0	0.1131	6.88E6
2QJB	AB	C	0.1	280000.0	0.0737	1.49E6
1A22	A	B	0.05	300000.0	0.0254	2.32E5
2QJA	AB	C	0.1	300000.0	0.0809	1.98E6
4HRN	A	D	0.1	322000.0	0.0459	5.12E5
3BT1	A	U	0.1	398000.0	0.0669	1.15E6
2QJ9	AB	C	0.1	400000.0	0.0964	3.61E6
2GOX	A	B	0.1	404000.0	0.1117	6.52E6
1REW	AB	C	0.1	600000.0	0.0934	3.21E6
2DSQ	I	G	0.1	671000.0	0.1269	1.17E7
1KTZ	A	B	0.16	740000.0	0.0818	2.05E6
1GL1	A	I	0.11	800000.0	0.0511	6.26E5
1X1X	A	D	0.1	897000.0	0.101	4.31E6
2FTL	E	I	0.1	990000.0	0.0135	1.46E5
3D5R	A	C	0.1	1200000.0	0.133	1.48E7
5XCO	A	B	0.1	1300000.0	0.0387	3.87E5
3D5S	A	C	0.1	1330000.0	0.1053	5.09E6
3BK3	A	C	0.1	1400000.0	0.0601	8.86E5
4UYQ	A	B	0.1	2000000.0	0.0497	5.93E5
3S9D	A	B	0.1	2100000.0	0.0606	9.03E5
4UYP	A	D	0.1	2100000.0	0.0837	2.21E6
1TM1	E	I	0.1	5600000.0	0.0499	5.97E5
2SIC	E	I	0.1	6500000.0	0.0038	1.00E5
1LFD	B	A	0.03	7700000.0	0.0591	8.53E5
1IAR	A	B	0.1	13000000.0	0.1628	4.71E7
2GYK	A	B	0.1	17300000.0	0.1652	5.17E7
1FR2	A	B	0.1	26000000.0	0.141	2.03E7
2VLO	A	B	0.1	34200000.0	0.1335	1.52E7
4G0N	A	B	0.1	35500000.0	0.1484	2.70E7
1B2U	A	D	0.1	37000000.0	0.0884	2.65E6
1B2S	A	D	0.1	43000000.0	0.0637	1.02E6
2WPT	A	B	0.1	50000000.0	0.2418	1.00E9
2VLP	A	B	0.1	65200000.0	0.1174	8.13E6
1EMV	A	B	0.25	72400000.0	0.1252	1.10E7
2VLQ	A	B	0.1	79100000.0	0.1913	1.42E8
2VLN	A	B	0.1	100000000.0	0.1819	9.86E7
1BRS	A	D	0.103	250000000.0	0.1166	7.88E6
1UEA	B	A	0.23	200000.0	0.0321	3.00E5
1QA9	A	B	0.166	400000.0	0.0933	3.20E6
2B4J	AB	C	0.181	480000.0	0.049	5.77E5
1SGN	E	I	0.26	1200000.0	0.0085	1.20E5
1VFB	AB	C	0.15	1400000.0	0.0248	2.26E5
1FLE	E	I	0.25	3600000.0	0.0649	1.07E6
1TLU	A	B	0.01	5600000.0	0.1007	4.26E6
1FFW	A	B	0.15	62000000.0	0.1588	4.03E7
4HTC	HL	I	0.175	75000000.0	0.1418	2.09E7
1UDI	E	I	0.08	150000000.0	0.226	5.43E8
2PCF	B	A	0.1	180000000.0	0.1769	8.12E7
7CEI	A	B	0.25	760000000.0	0.1611	4.41E7
5F4E	A	B	0.1	115000.0	0.0222	2.05E5
3BP8	A	C	0.223	995000.0	0.011	1.33E5
1JTG	A	B	0.025	108000.0	0.0427	4.52E5
1SBB	A	B	0.16	100000.0	0.0037	1.00E5
4HSA	AB	C	0.1	246000.0	0.073	1.46E6
1EWY	A	C	0.31	40000000.0	0.0651	1.08E6
1OC0	A	B	0.165	14000000.0	0.0859	2.40E6
1AGR	A	E	0.11	1700000.0	0.0282	2.58E5

Table S1
